# Animal models of Takotsubo syndrome: bridging the gap to the human condition

**DOI:** 10.3389/fcvm.2024.1351587

**Published:** 2024-05-22

**Authors:** Ermir Zulfaj, AmirAli Nejat, Abdulhussain Haamid, Ahmed Elmahdy, Aaron Espinosa, Björn Redfors, Elmir Omerovic

**Affiliations:** ^1^ Department of Molecular and Clinical Medicine, Institute of Medicine, Gothenburg University, Gothenburg, Sweden; ^2^ Department of Cardiology, Sahlgrenska University Hospital, Gothenburg, Sweden; ^3^ Core Facilities - Experimental Biomedicine, Sahlgrenska Academy, Gothenburg, Sweden

**Keywords:** Takotsubo syndrome, animal model, translational research, heart failure, stress cardiomyopathy

## Abstract

Modelling human diseases serves as a crucial tool to unveil underlying mechanisms and pathophysiology. Takotsubo syndrome (TS), an acute form of heart failure resembling myocardial infarction, manifests with reversible regional wall motion abnormalities (RWMA) of the ventricles. Despite its mortality and clinical similarity to myocardial infarction, TS aetiology remains elusive, with stress and catecholamines playing central roles. This review delves into current animal models of TS, aiming to assess their ability to replicate key clinical traits and identifying limitations. An in-depth evaluation of published animal models reveals a variation in the definition of TS among studies. We notice a substantial prevalence of catecholamine-induced models, particularly in rodents. While these models shed light on TS, there remains potential for refinement. Translational success in TS research hinges on models that align with human TS features and exhibit the key features, including transient RWMA. Animal models should be comprehensively evaluated regarding the various systemic changes of the applied trigger(s) for a proper interpretation. This review acts as a guide for researchers, advocating for stringent TS model standards and enhancing translational validity.

## 1 Introduction

Modelling human disease is invaluable for gaining new insights into the mechanisms underlying organ function and establishing the pathophysiology of human diseases. Takotsubo syndrome (TS) is an acute form of heart failure with mortality and clinical presentation similar to that of myocardial infarction (1). It is recognized by reversible regional wall motion abnormalities (RWMA) in the ventricles. Several hypotheses have been proposed to explain this syndrome, but they are inconsistent, surrounded by controversies, and leave many questions unanswered, resulting in a lack of evidence-based treatments specifically for TS (2, 3). There seems however to be a consensus regarding the pivotal role of catecholamines in the disease (2).

The main impediment to creating a TS model is the lack of an appropriate understanding of the pathophysiology. However, several critical characteristic phenotypes have been described (4), and based on these notions, animal models of TS have been developed (5–16).

In this article, we aim to review and evaluate current animal models of TS, focusing on their ability to replicate essential clinical characteristics observed in human patients and identifying their limitations. Through this review, we will summarize the existing animal models and highlight the crucial features of TS.

## 2 Clinical characteristics of Takotsubo syndrome

Although treatment trials are underway (17), there are currently no evidence-based treatment options for TS. For clinicians, differentiating TS from other cardiac diseases poses a great challenge, often leading to unnecessary invasive procedures and a high demand for the development of diagnostic markers.

### 2.1 History

Our knowledge and understanding of TS have developed over the years, along with its definition, disease classification, and nomenclature. TS was first described in the early 1990s (18). It was initially named “takotsubo” due to its characteristic shape and appearance during contraction, resembling a “Japanese octopus fishing pot”. However, the disease as an entity is believed to have been recognized earlier (19). The disorder later received the name “stress-induced cardiomyopathy”, owing to the importance of stress as its trigger. It was classified as both a primary and acquired cardiomyopathy by the AHA in 2006 and as an unclassified cardiomyopathy by the ESC in 2007. Moving forward, we will use the term TS, acknowledging that its definition is based on clinical observations (20).

### 2.2 Epidemiology

TS manifests as an acute form of heart failure that typically begins suddenly and is frequently triggered by stress, either emotional or physical, which is identified in approximately two-thirds of patients. Not everyone develops TS following a stressful event, suggesting that a variety of factors influence its development. Some reported factors are related to thyroid function (21), climatic and body temperatures (22, 23), malignancy (24), as well as age, sex, and neurological and psychiatric disorders.

The incidence of TS increases with age for both men and women, with a notably steeper rise occurring among women of perimenopausal age. While TS can affect individuals of any age and sex, it predominantly occurs in post-menopausal women, who represent approximately 80% of all reported cases. In comparison, TS is more frequently diagnosed in older men than in premenopausal women and is relatively rare among younger individuals, irrespective of their sex (25). This pattern underscores that TS is a syndrome of the postmenopausal woman, rather than women in general. The predisposing mechanism of age has been speculated to be associated with the increase in resting sympathetic nervous activity observed with aging (26). An age-related effect of hypogonadism, with oestrogen deficiency being more important than testosterone deficiency, have also been suggested (27).

Patients with TS frequently exhibit major depressive disorders and generalized anxiety disorder, indicating the potential role of psychosocial factors as predisposing risks in the pathophysiology of TS (27, 28). This link is supported by the fact that regions in the limbic system, which regulate sympathetic activation and stress response, are closely associated with anxiety and depression (29). Disturbances in these limbic regions could influence the stress response, thereby contributing to the development of TS (30). This theory is further corroborated by reports that associate neurologic disorders with TS, underscoring the interconnectedness of psychological and neurological factors in its onset (25, 31).

### 2.3 Diagnostic criteria

Several diagnostic criteria have been proposed, and new and updated criteria are continually being released. The latest diagnostic criteria, the InterTAK Diagnostic Criteria, was published in 2018 (4). These criteria are based on clinical observations and mainly relate to morphological changes, the natural course of the condition, the presence of myocardial injury, and the exclusion of other diagnosis. Additional features have also been described to assist clinicians in making the diagnosis. We will consider these characteristics as we evaluate current animal models of TS.

#### 2.3.1 Morphological changes and natural course

One of the most characteristic features of TS is the presence of transient RWMA. The observed RWMA can be localized in both ventricles and affect any segment. The typical pattern, seen in 80% of cases, involves symmetrical involvement of the mid and apical segments of the left ventricle with relatively preserved basal segments, resulting in the characteristic “apical ballooning”. Other variants include only mid-ventricular segments, basal segments alone (i.e., reversed TS), or even a focal region. Cardiac function and RWMA typically recover almost entirely within a few days to weeks if the patient survives the acute phase (4). The various diagnostic criteria proposed for TS differ in certain aspects, yet they all emphasize the importance of observing RWMA and its resolution. In clinical practice, cardiac imaging is thus essential for diagnosing TS, as conclusively identifying the condition without it is not feasible. In this regard, we consider this characteristic of transient RWMA to be an essential feature of TS. It is important to note that the recovery of RWMA is a part of its natural course and should be present. Echocardiography, the most readily available and commonly used imaging technique, is often employed to assess changes in left ventricular function. Other techniques include cardiac magnetic resonance (CMR) imaging, cardiac computed tomography angiography (32), and ventriculography. The latter can be combined with coronary angiography, which is necessary for excluding acute coronary syndrome as a cause of RWMA. In patients with acute TS, the typical pattern observed on CMR includes RWMA, localized myocardial oedema, absence of significant necrosis or fibrosis, and markers of inflammation, such as increased early myocardial uptake of gadolinium (33). This inflammation is primarily mediated by innate immune cells during the acute phase. Histological observations also include disturbances in protein metabolism, accumulation of lipid droplets and glycogen deposits, contraction bands, and a temporary reduction in contractile proteins (16, 34). The importance of these changes for the development of RWMA remains unknown. Cardiac nuclear imaging techniques have been used in TS for assessment of perfusion, metabolism, and innervation, but they do not directly diagnose the RWMA. It is important to note that the mere presence of transient RWMA alone is not sufficient for a definitive TS diagnosis.

#### 2.3.2 Myocardial injury and exclusion of other diagnosis

The necessity of myocardial injury, verified either by new electrocardiogram (ECG) changes or the release of cardiac biomarkers, for diagnosing TS varies across different criteria. Neither ECG changes nor cardiac biomarkers possess clinically meaningful sensitivity or specificity for TS (25). Despite this, most of the proposed diagnostic criteria consider them either mandatory or optional, attributing this to their strong association with TS. Over 80% of patients with TS present with elevated troponin values, and nearly the same percentage show new changes in their ECG at presentation (25). Consequently, it is quite rare to encounter a TS patient without elevated troponins or new ECG changes, especially with the introduction of more sensitive assays. The cardiac biomarker profile in TS is characterized by a modest elevation in troponin and a more significant increase in N-terminal prohormone of brain natriuretic peptide (NT-proBNP) compared to acute myocardial infarction (35, 36). Although the levels of troponin and NT-proBNP vary among laboratories, the median troponin T peak in TS patients has been reported in different studies. Patients with acute myocardial infarction exhibit troponin peaks that are 6–15 times higher and NT-proBNP values that are 2.4–3 times lower than those in TS patients (35, 37).

The ECG changes in TS are dynamic and bear a resemblance to those observed in acute myocardial infarction, exhibiting a distinct progression. Initially, any ST-segment elevation, present in about 50% of cases (32, 38), tends to resolve. This is followed by a biphasic temporal pattern where T-wave inversion and QT interval lengthening occur over several days (39). Eventually, these changes gradually diminish, with both the T-wave inversion and the QT interval prolongation resolving over time (38). The typical ECG changes described for TS include deep symmetrical T-wave inversion in the anterior leads with QT prolongation (36), similar to the ECG pattern seen in Wellen's syndrome. Other less common changes, such as ST-depression and pathological Q-waves, can also be observed.

The diagnosis of TS is contingent upon the presence of crucial features such as transient RWMA, ECG changes, and elevated cardiac troponin. However, a definitive diagnosis of TS also relies on ruling out other potential causes of these symptoms, with coronary obstruction and myocarditis being the most common. The concept of TS as a diagnosis of exclusion is a consistent element across all diagnostic criteria. While these exclusion criteria vary in their range of diseases, more recent diagnostic criteria have become less stringent, featuring fewer contraindications. This more flexible approach further emphasizes TS as a condition characterized by transient RWMA. Therefore, when modelling the disease, this feature is essential and cannot be overlooked. By incorporating clinically relevant triggers into the model, we acknowledge TS as a diagnosis of exclusion.

### 2.4 Complications and predictive factors

The reversible nature of TS often leads to the perception that it is a benign disease, but its in-hospital mortality rate of 4%–5% is comparable to that of acute myocardial infarction (1). Higher mortality rates, between 7%–13%, have also been reported in some studies (40, 41). Additionally, TS is associated with relatively high complication rates, especially during the acute phase, with up to two-thirds of patients experiencing complications. The most common complication is heart failure, followed by left ventricular outflow tract obstruction, mitral regurgitation, and cardiogenic shock. Other less frequent complications include the formation of apical thrombi in the left ventricle and arrhythmias (32, 41). Predictive factors for complications or worse outcomes include physical trigger, older age, comorbidities, higher troponin levels, and reduced systolic cardiac function (25, 42). While male sex is often significant in univariate analyses, its significance tends to diminish in multivariate analyses (25). High body temperature at admission has been shown to be a strong predictor of poor outcomes in patients with TS (43). Moreover, several retrospective studies suggest that TS may carry a worse long-term prognosis, often with persistent abnormalities in cardiovascular function (44, 45). It has been reported that systemic inflammation can persist for at least five months (46). While there is typically no evidence of macroscopic fibrosis on CMR, diffuse microscopic fibrosis has been detected (47). The recurrence rate of TS is approximately 5%. A significant number of these recurrences exhibit a different TS pattern from the initial episode, suggesting a possible protective effect of previous TS episodes on the affected region (48). Additionally, neurological and psychiatric disorders have been identified as independent predictors of recurrence (49).

### 2.5 Potential etiological mechanisms

The precise mechanisms underlying TS development are still unknown, with several pathophysiological theories proposed. These theories are broadly categorized into myocardial oxygen imbalance and direct myocardial impact, as outlined in Figure 2. When evaluating the validity of these mechanisms, it is important to consider the diverse clinical manifestations of TS. Myocardial oxygen imbalance involves a mismatch between supply and demand. Diminished supply may arise from coronary artery spasms or microvascular dysfunction, while heightened demand may stem from increased wall stress, either globally or in specific regions (50). Direct myocardial impact is concerned with the effects of β-receptor overstimulation. Typically, stimulation of β-receptors leads to an increased inotropic effect through a signal transduction cascade involving G-protein-coupled receptors and the generation of second messengers like cyclic adenosine monophosphate. These messengers play a crucial role in calcium handling and contractile function. However, overstimulation of this pathway can lead to reduced contractility and cell death due to impaired calcium handling (51, 52). As a protective response, it has been proposed that TS may develop from a negative inotropic effect on myocyte contraction, attributed to a shift in the β2-adrenoreceptor subunit (53). The negative inotropic effect is expected to be most pronounced at the heart's apex, where β-receptor density is believed to be highest. However, this hypothesis faces a challenge when considering the non-apical variants of TS observed both across different patients and within the same individual.

## 3 Animal models of Takotsubo syndrome

Models of TS serve two distinct aims, each contributing valuable insights. The first aim is to provide mechanistic understanding that is not easily attainable in clinical settings. To achieve this, experimental studies often employ reductionistic approaches that may have limited direct applicability to the clinical situation. The second aim is to bridge the gap between experimental findings and the clinical scenario, necessitating models that closely replicate the clinical setting. In this review, our primary focus is on models that align with the latter aim, aiming to enhance translation to the clinical context. However, it is important to recognize the significance of both types of models.

Various frameworks for assessing the quality of an animal model have been established (54–57). These frameworks generally evaluate the relevance of the species used and the extent to which the model replicates the human condition. In this review, our primary focus will be on the latter aspect. We will however briefly mention that it is commonly assumed that the closer a species is to humans, the more likely it is to exhibit similar disease pathophysiology. Due to practical and ethical considerations, as well as the availability of genetic modification techniques, small animals are frequently employed. Small animals have been the primary choice for cardiovascular research for many years, and their findings have demonstrated successful translation to humans (58). This success is largely due to the similarities in cardiovascular physiology between small animals and humans. However, it is important to note that significant differences do exist, including variations in total heart size, physiological heart rate, and electrophysiological differences, among others. The specific key features required for the development of TS in humans remain unknown. However, the observation that stress can induce similar characteristic apical ballooning in several species during experimental setups suggests that these features might be shared across different species. Furthermore, it is important to acknowledge that not all individuals develop TS in response to stress, highlighting the role of individual variability and the importance of environmental factors. In this context, utilizing outbred animals (i.e., non-gene modified) with a variable genetic profile recognizes that genetics confer risk but not certainty.

### 3.1 Brief history

Effects of stress on the heart have been reported in scientific research as early as 1906 (59), and since then, an increasing number of reports have utilized catecholamines to study cardiac injury and lesions (60). Reports during the 1970s demonstrated similar cardiac lesions in humans following subarachnoid hemorrhage (61), pheochromocytoma (62), and in victims of assault (63), resembling the findings observed in rats after systemic administration of catecholamines. These findings underscored the significance of experiments involving the exogenous administration of catecholamines and contributed to the coining of the term “stress cardiomyopathy” (63). In 2002, Ueyama et al. introduced the first animal model of TS (8). They achieved this by restraining a rat on its back for 30 min, inducing intense emotional stress as evidenced by elevated plasma catecholamine levels. This experimental setup led to the observation of the characteristic phenotype of apical ballooning during left ventriculography. A few years later, a model of pilocarpine-induced epilepsy was reported to induce apical akinesia or dyskinesia after 2 h (64), and the infusion of epinephrine was reported to induce “takotsubo-like apical akinesia” in non-human primates (9). Since then, the administration of exogenous catecholamines has become the predominant method for modelling TS.

### 3.2 Discrepancy of TS definition

**Figure 1:**
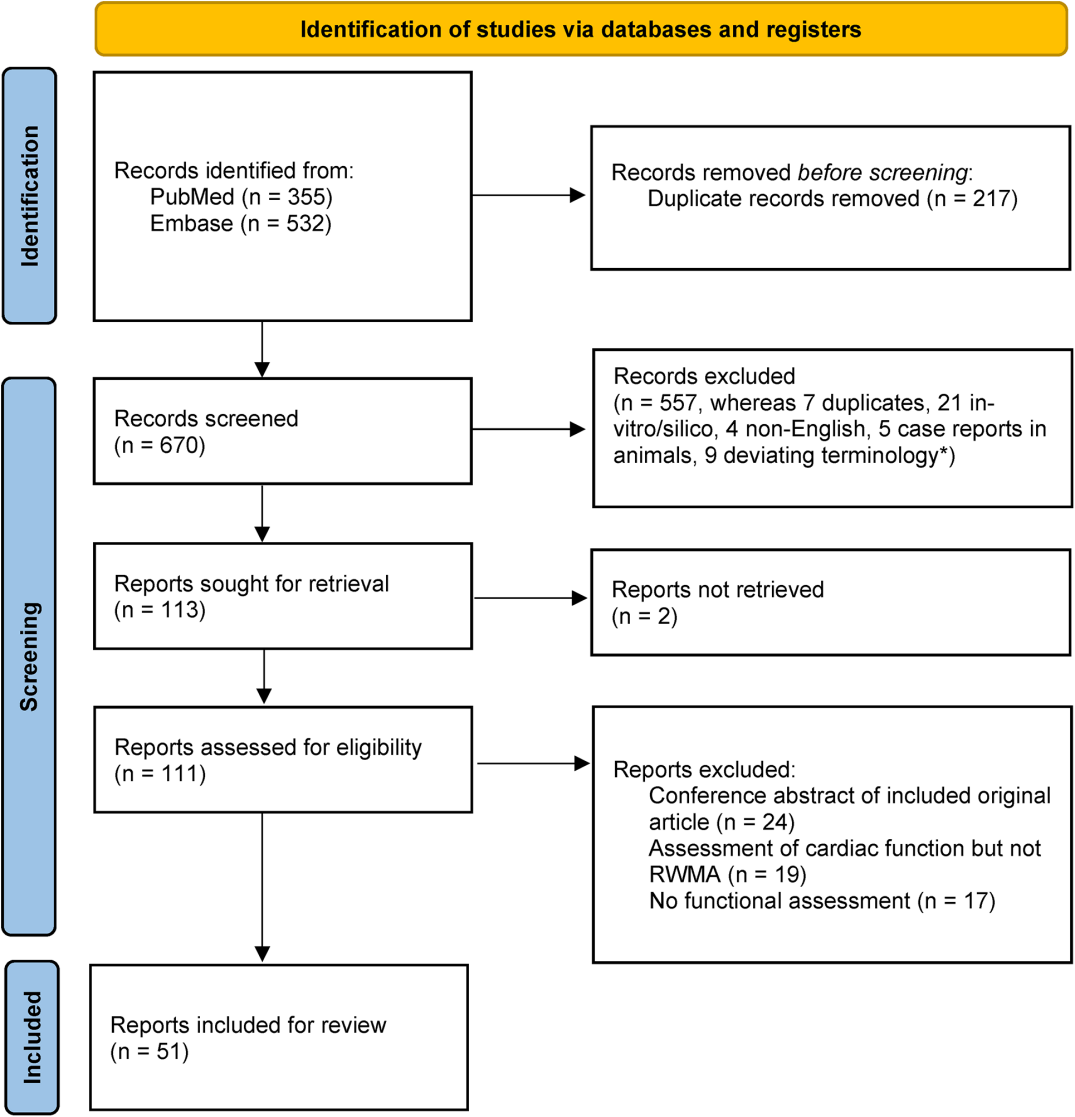
PRISMA 2020 flow diagram (65). *Terminology of stress-induced cardiomyopathy was used although not implying Takotsubo syndrome.

**Figure 2:**
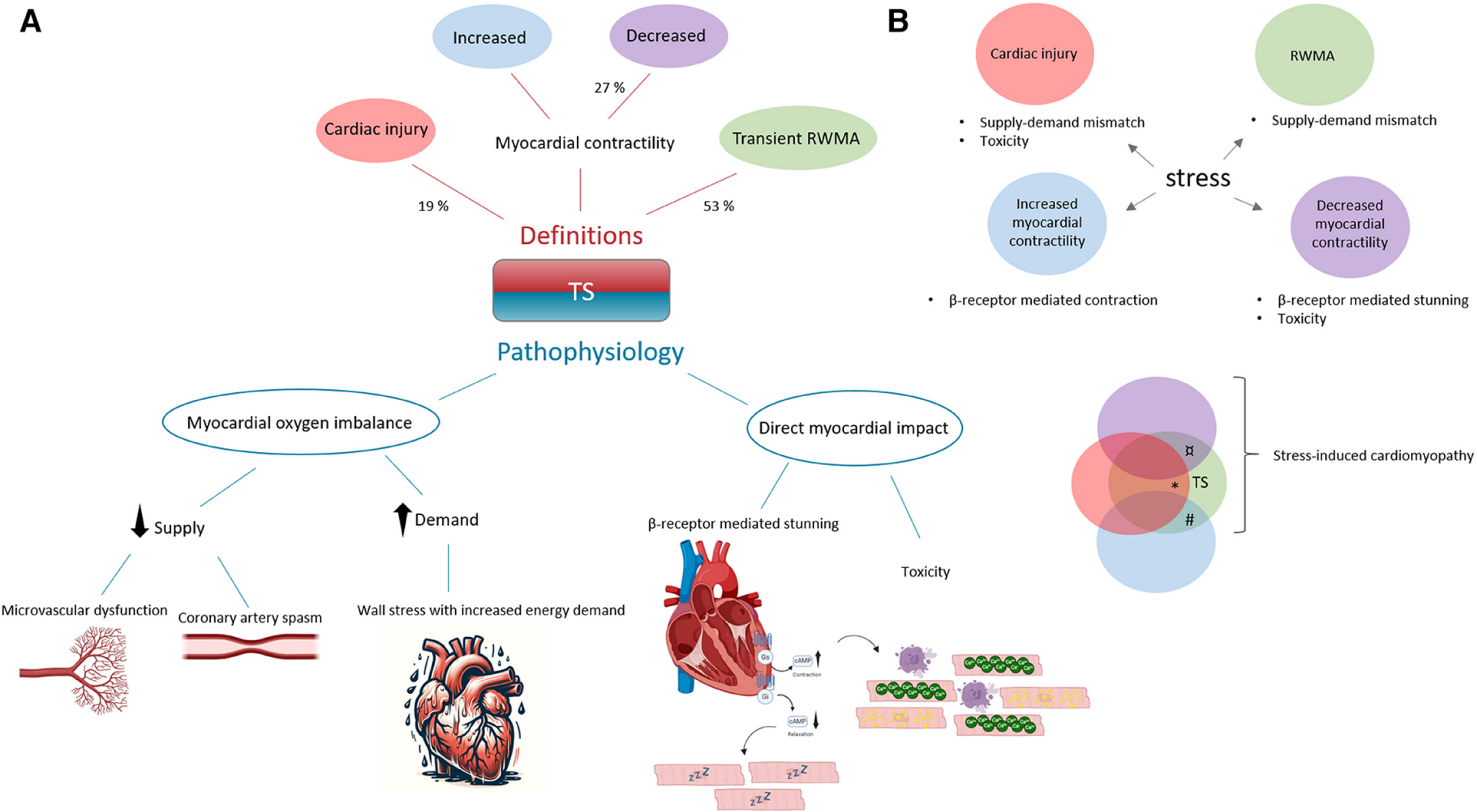
A comprehensive view of the definitions and underlying mechanisms in animal models of Takotsubo syndrome (TS), with respective proportions (*n* = 87). (**A**) In animal studies, definitions of TS are largely based on the occurrence of cardiac injury (19%), reduced myocardial contractility (27%), or transient regional wall motion abnormalities (RWMA, 53%)—with the latter, marked in green, being a hallmark of TS. The pathophysiological mechanisms are categorized into two types: those related to an imbalance between myocardial oxygen supply and demand, and those arising from direct myocardial effects due to excessive β-receptor stimulation. (**B**) The phenotypes highlighted here reflect the range of cardiac manifestations to stress, which can occur either in isolation or in combination. We offer a potential explanation for the variable clinical presentations and outcomes observed in TS. For example, extensive cardiac injury, denoted in red*, may trigger a strong inflammatory response, leading to worse long-term outcomes. An obstruction in the left ventricular outflow tract could develop as a consequence of a hypercontractile base, shown in blue#. Furthermore, reduced myocardial contractility, indicated in purple¤, is a predictor of worse prognoses.

The method for our search strategy can be found in the Supplementary Material. A total of 111 reports, comprising original articles and conference abstracts, were retrieved and evaluated for eligibility [the corresponding PRISMA 2020 flow diagram (65) can be found in Figure 1]. Among these, 24 were excluded due to the duplication of conference abstracts in subsequently published original articles, although some overlap might still exist. The remaining 87 reports encompass the entirety of animal models employed in TS research. During the screening process, we observed variations in the definition of TS. TS was defined based on various potential manifestations of a stressful trigger, encompassing measures of cardiac injury such as histological findings and biomarkers, as well as the mere presence of a stressor (19% of the reports). Alternative definitions focused on changes in global myocardial contractility or in specific regions only (27%) and assessment of RWMA (53%) (Figure 2). Among these three definitions, it is worth noting that the current criteria for diagnosing TS include only the assessment of RWMA and cardiac biomarkers. Since the release of cardiac biomarkers is highly unspecific for distinguishing cardiac conditions, we included reports that assessed for RWMA as a minimum requirement for further review. A possible reason why nearly half of the reports did not include the assessment of RWMA in their definition of TS may stem from the use of the more general term “Stress-Induced Cardiomyopathy” (SIC). This ambiguous terminology, possibly combined with the complex relationship between stress, cardiac dysfunction, and cardiac injury, could contribute to the observed variations. Nine records that were excluded during the first screening used the terminology of stress cardiomyopathy, although not implying to TS. These records employed this terminology to study the effects of reductive stress (66), exertional heat stroke (67), subarachnoid haemorrhage (68), settings of catecholamine excess (69), physiological disturbances (70), toxicity (71), etc., on the heart. However, while both subarachnoid haemorrhage and exogenous catecholamine administration can be used as induction methods for a TS model, their presence does not necessarily implicate TS. For the same reason, studies conducted in the 1900s on the effects of exogenous catecholamine administration on the heart should not be regarded as models of TS. We believe that SIC could be used as a hypernym for stress-induced changes in the heart, and, as is apparent from the clinical setting, it is important to distinguish the transient loss of cardiac function in a cardiac region occurring after stress (Figure 2).

Among the remaining 51 reports, assessing wall motion abnormalities was a common focus. However, we observed variations in the specific endpoints studied and, importantly, in distinguishing observations with a TS phenotype. Out of the 51 reports, 34 considered RWMA and identified observations with a TS phenotype, representing less than half of all TS research utilizing animal models. The remaining 17 reports either did not attempt to distinguish the TS phenotype (including animals with and without the TS phenotype) or examined changes in contractility compared to the baseline. While it is important to consider baseline measurements, comparing changes in contractility to the baseline presents limitations in clinical relevance and fails to account for regional differences within the ventricle. This may explain why a significant change in this endpoint does not always correlate with a significant change in phenotype. Several reports included some form of measure of systolic cardiac function as a primary endpoint. Global cardiac function is an important outcome measure in TS, as it has been identified as an independent predictor of worse outcomes (25). However, for the study to be clinically applicable, it is crucial that global cardiac function, when used as an outcome measure, be evaluated in the context of a TS phenotype. The impact of stress inducing a reduced global systolic function, in the absence of RWMA, appears to be a recurring finding observed by us and others (8, 10, 72). Thus, regions with wall motion abnormalities can be associated with segments displaying hyperkinesia, no change, or even hypokinesia. This variation in contractile segments contributes to a variable exaggerated ballooning effect, potentially indicating a condition distinct from the RWMA observed in TS and characterized by different pathomechanisms. For example, the akinetic regions could be primarily driven by a myocardial oxygen imbalance, while the hypokinetic and hyperkinetic regions depend on beta-adrenergic receptor signalling. It is worth noting that global left ventricle hypokinesia, although proposed as a potential manifestation of TS (73) and an important cardiac state that should not be disregarded, is not considered a defining feature of TS due to its broad differential diagnosis. This is consistent with the diagnostic criteria, which emphasize the importance of pattern (i.e., regionality) in identifying TS (4). We propose a model to explain how stress can induce various manifestations through potentially different pathophysiological mechanisms (Figure 2). This model provides a potential explanation for the variable clinical presentations and outcomes seen in TS, emphasizing the complex nature of TS and the importance of precise definitions in preclinical research.

### 3.3 Translational aspects

A summary of the current published models of TS is presented in Table 1. The most widely utilized model, accounting for 85% of all publications, involves the exogenous administration of catecholamines. Isoprenaline is the catecholamine most frequently used, employed in 79% of cases, and is predominantly administered intraperitoneally (IP) in 87% of studies. The IP administration of isoprenaline, which reproduces the RWMA as reported in patients, was first introduced by the Omerovic Lab (Gothenburg, Sweden) in 2013 in mice (16) and later in rats (14). The TS-like RWMA has also been reproduced in non-human primates (9) and rabbits (74). However, the most commonly used animal for TS research is the naïve rat (no pre-treatment or genetic modification), accounting for 72% of studies.

**Table 1:** Models of Takotsubo syndrome categorised by trigger and animal (51 reports).

Transaortic constriction on gene-modified mouse (74, 75)
Pilocarpine-induced epilepsy in rat (64, 76)
High dose of catecholamine in:
• Mouse (16, 72, 77–85) • Rat (11–15, 23, 86–103) • Monkey (9) • Gene-modified mouse (104) • Subarachnoid haemorrhage in rabbit (105)
High dose of PDE-3 inhibitor in rat (106)
Low-moderate dose of catecholamine in:
• Rat (10, 107, 108) • Transfected rat (109) • Refined model in rat (110)
Immobilization in rat (8, 111, 112)

Doctor Scantlebury and Prasad neatly summarized the proposed diagnostic criteria and features of TS in their table 3 (113). In our Table 2, we present a modified version of this, summarizing the key characteristics of TS and associated features observed in humans, alongside the InterTAK criteria, and illustrating how different models of TS replicate these features. A model that reproduces all of these characteristics would indicate a very high degree of construct validity. Our aim is to highlight the features of TS, shed light on the strengths and limitations of each model, and identify possible actions to bridge the gap for successful translation. This initial step in model evaluation can be seen as “reverse translation”, and if a model demonstrates high construct validity, it is reasonable to assume that the pathophysiology is also accurately represented in the model. A strong model provides a platform for both translational research and the investigation of pathophysiological mechanisms.

**Table 2:** Features of Takotsubo syndrome. Bolded features correspond with mandatory clinical criteria. ‘✓': Replicated; ‘✗’: Not replicated; ‘?': Not studied or reported; ‘NA': Not applicable. InterTAK Diagnostic Criteria: ‘m' for mandatory, ‘o' for optional, ‘x’ not explicitly mentioned for diagnosis. Abbreviations: Iso, Isoprenaline; Epi, Epinephrine; Norepi, Norepinephrine; SAH, Subarachnoid Hemorrhage; TAC, Transverse Aortic Constriction.

Diagnostic criteria (4, 20, 113)	Refined TS-model in rat (110)	Iso IP (rat (11–14, 23, 86–103) / Mouse (16, 72, 79–84))	Iso SC (rat (15)/ mouse (77, 78, 85))	Epi rat (IV (10, 120, 121)/IP (11))	Restrained rat (30 (8, 112)/120 min (111))	Kv-/- mouse (TAC (74, 75)/Norepi (104))	SAH in rabbit (105) /epilepsi in rat (64, 76)	Epi IV monkey (9)	InterTAK (4)
- Morphology									
**Regional wall motion abnormality (RWMA)**	✓	✓/ ✓	✓/ ✓	✗/ ✓	✓/✓	✓/✓	✓/✓	✓	m
…extending beyond a single epicardial vascular distribution (or circumferential pattern)	✓	✓/✗	✓/✗	?/?	✓/?	✓/✓	?/ ✓	?	o
Involvement of apical with mid-ventricular segments (classic apical ballooning pattern)	✓	✓/✗	✓/✓	?/?	✓/✗	✓/✓	?/ ✓	?	o
Atypical TS (midventricular, basal, focal)	?	✓/✓	✗/✓	?/ ✓	?/ ✓	?/?	?/?	?	o
Right ventricular wall motion abnormalities	?	?/?	?/?	?/?	?/?	?/?	?/?	?	o
- Time course of RWMA									
**“Transient”**	✓	✓/ ✓	✓/?	?/?	✓/?	✗/?	✓/✓	✓	m
(Near) Complete recovery within days to weeks	✓	✓/✓	?/ ✗	?/?	✓/?	✗/?	✓/✓	✓	o
- Evidence of ischemia/myocardial injury									
**New and dynamic ST-segment deviation, QT prolongation, T-wave inversion and/or left BBB**	✓	✓/✓	?/?	✓/✓	✓/?	?/?	✓/✓	✓	o
**‘Mild’ or ‘modest’ increase in cardiac biomarkers**	elevated	?/elevated	?/elevated	?/?	?/elevated	?/?	?/?	?	m
Disparity between the troponin level and the amount of the dysfunctional myocardium present	?	?/?	?/?	?/?	?/?	?/?	?/?	?	x
- Exclusions									
**Potential coronary culprit (e.g., stenosis, evidence of plaque rupture, dissection, thrombosis or spasm)**	✓	✓/✓	✓/✓	✓/✓	✓/✓	✓/✓	✓/✓	✓	m
Myocarditis	✓	✓/✓	✓/✓	✓/✓	✓/✓	✓/✓	✓/✓	✓	m
Other pathological conditions that may explain regional dysfunction	✓	✓/✓	✓/✓	✓/✓	✓/✓	✗/?	✓/✓	✓	x
- Other features									
Symptoms similar to that of acute coronary syndrome	NA	NA	NA	NA	NA	NA	NA	NA	x
Elderly patient	?	?/?	?/?	?/?	?/?	?/?	?/?	?	x
Postmenopausal woman	?	?/?	?/?	?/?	?/?	?/?	?/?	?	o
Antecedent stressful event	✓	✓/✓	✓/✓	✓/✓	✓/✓	x/✓	✓/✓	✓	o
Normal or near normal filling pressures	?	✓/?	✓/?	?/?	?/?	?/?	?/?	?	x
Abnormal myocardial scintigraphy	?	?/?	?/?	?/?	?/?	?/?	?/?	?	x
Mortality (emotional 1%, physical 7–13%) (4)	✓	✗/✗	✗/?	?/?	?/?	?/?	?/?	✗	x
Psychiatric disorders (25)	?	?/?	?/?	?/?	?/?	?/?	?/?	?	x
Reocurrent (32, 48)	✓	?/ ✓	?/?	?/?	?/?	?/?	?/?	?	x
Complication profile (32)									
Acute heart failure	✓	✓/✓	✓/✓	?/ ✓	✓/✓	✓/✓	?/?	✓	
LVOT	?	?/?	?/?	?/?	?/?	?/?	?/?	?	
Mitral regurgitation	?	?/?	?/?	?/?	?/?	?/?	?/?	?	
Cardiogenic shock	✓	✓/✓	?/?	?/ ✓	?/?	?/?	?/?	?	
Atrial fibrillation	?	?/?	?/?	?/?	?/?	?/?	?/?	?	
Ventricular tachyarrythmia	✓	✓/✓	?/?	✓/✓	?/?	?/?	?/?	✓	
bradyarrhythmia	✓	✓/✓	?/?	?/?	?/?	?/?	?/?	✓	
Thrombus formation	✓	?/?	?/?	?/?	?/?	?/?	?/?	?	
Predictive factors (25, 32, 42, 43)									
Systolic cardiac function	✓	?/?	?/?	?/?	?/?	?/?	?/?	?	
Body temperature	?	?/?	?/?	?/?	?/?	?/?	?/?	?	
Trigger	✓	?/?	?/?	?/?	?/?	?/?	?/?	?	
Cardiac troponins	?	?/?	?/?	?/?	?/?	?/?	?/?	?	
Treatment	?	?/?	?/?	?/?	?/?	?/?	?/?	?	
Age	?	?/?	?/?	?/?	?/?	?/?	?/?	?	
Comorbidities (malignancy, kidney disease, neurologic disorders etc.)	?	?/?	?/?	?/?	?/?	?/?	?/?	?	

When observing Table 2, several aspects stand out. First, there is a set of features considered crucial for diagnosing TS. As previously mentioned, they relate to the presence of RWMA, the transient course, the exclusion of myocardial infarction and myocarditis, ECG changes and the release of cardiac biomarkers. For an animal model to be relevant in translational research for TS, these features must be present. Second, there is a need for further validation of the animal models. Many features are either not recognized or reported, underscoring the importance of presenting negative findings as well. Table 2 is available for researchers to validate their models. If a non-crucial feature is not replicated, it does not invalidate a model but rather illuminates differences whose importance may not be immediately apparent. The features listed are not considered to be exhaustive and may need updates based on our evolving understanding of TS. The table can serve as a guide for future investigations. It is noteworthy that the role of hormonal status and age in the context of TS has not been extensively studied. The reason why approximately 80% of reported cases occur in postmenopausal women is unkown (25). While studies have been published on the effect of stress and hormonal status on cardiac function (76–78), they have not addressed the crucial features of TS. We would like to emphasize further that being a postmenopausal woman is a characteristic feature of TS, rather than a general trait applicable to all woman. This distinction appears to be a common misconception in TS modelling, where female animals are incorrectly assumed to be more relevant. Considering the clinical characteristics previously discussed, both females and males are suitable choices for studying the TS phenotype, i.e., apical ballooning. However, it is important to note that female rodents, whether young or aged, may not accurately represent postmenopausal women (79).

Critical aspects in the assessment of TS models include the induction trigger or experimental design, the transient nature, and acute onset. The use of exogenous catecholamines as a trigger in TS models has raised concerns due to their similarity to pheochromocytoma, previously a diagnosis excluded from TS. However, revised criteria now acknowledge pheochromocytoma as a potential TS trigger, emphasizing the importance of treating the underlying disorder (4, 20). Exogenous catecholamine administration has moreover been identified as a trigger for TS in patients. Notably, catecholamines can induce dose-dependent cardiac toxicity (80, 81), unrelated to the TS phenotype. Thus, for this trigger, the lowest effective dose that allows extensive apical akinesia with a high incidence and low mortality is recommended. This also highlights the need for positive controls, i.e., catecholamine-treated rats without TS development. The transient course further lacks a specific timeframe, recovery is generally described as days to weeks (4). Published animal models show variations, with some reporting recovery within minutes (10, 74) and others over hours to days (14–16, 82). The different diagnostic criteria do not specify the onset as either acute or chronic. To our knowledge, the development of TS over days or weeks has not been reported. TS is recognized as an acute form of heart failure, akin to acute coronary syndrome in its clinical presentation. We believe that accurately replicating an acute onset and recovery similar to that seen in patients is crucial for representing the essential pathophysiological mechanisms within the model. We would like to emphasize the importance of considering these aspects in model development by reviewing Dong et al. recent gene-modified mouse model of TS involving transaortic constriction (74). While their model induces apical akinesia resembling RWMA in TS, it overlooks crucial TS characteristics: acute onset, relevant trigger, and transient nature. Their model aligns more closely with “myocardial hibernation”, occurring in chronic supply-demand mismatch states (119). Molecular investigations in their study reveal gene expression similarities with hibernation, as noted by the authors. This connection suggests that hibernation, stunning, and RWMA in TS could partly share pathogenesis, supporting previous reports that link supply-demand mismatch as a key TS driver. Other induction methods for triggering TS include restraining, pilocarpine-induced epilepsy, the combination of intracranial blood infusion with norepinephrine, and a PD3-inhibitor (Table 1). The latter three induction methods require chemical agents which, similar to the catecholamine-induced models, might induce toxicity or physiological changes unrelated to the TS phenotype and need to be evaluated. In the first three induction methods, a systemic stress response plays a central role. The TS model that combines intracranial blood infusion and norepinephrine offers an important clinical scenario resembling subarachnoid haemorrhage (120). Although clinically relevant, this model provides a limited representation of TS, characterized by a sparse akinetic region and a duration of only 15 min (105). Both restraining and pilocarpine-induced epilepsy results in an endogenous increase of catecholamines and a TS phenotype that recovers within 24 h. Inducing TS by physically restraining an awake animal offers a stress response without drug administration but raises significant ethical considerations. Mortality numbers in such models have not been presented, however, an incidence of 40%–90% from two different labs has been reported, with the onset described as acute (30 min) and accompanied by troponin release and new ECG-changes (8, 111). The pilocarpine-induced epileptic seizure could serve as a relevant trigger (121). The model have a reported incidence of 67%, featuring an acute onset ranging from 30 to 120 min, and accompanied by new ECG-changes (76). However, mortality data are lacking. Models applying a PD3-inhibitor aim to induce TS by circumventing the process of catecholamine ligand binding and β-receptor activation. High-dose administration of a PD3-inhibitor in rats has led to apical ballooning after 90 min, resembling the RWMA in TS (106). A mortality rate of 30% and an incidence rate of 20% were reported in this setting, although essential TS features such as a transient course and ECG-changes were not mentioned. Whether bypassing catecholamine ligand binding and β-receptor activation actually occurred in the model was not verified, which is most likely considering that a PD3-inhibitor can increase norepinephrine release (122). Many TS models found in the literature review lack a proper evaluation of the TS features observed in humans (Table 2).

### 3.4 Modelling TS moving forward

**Figure 3:**
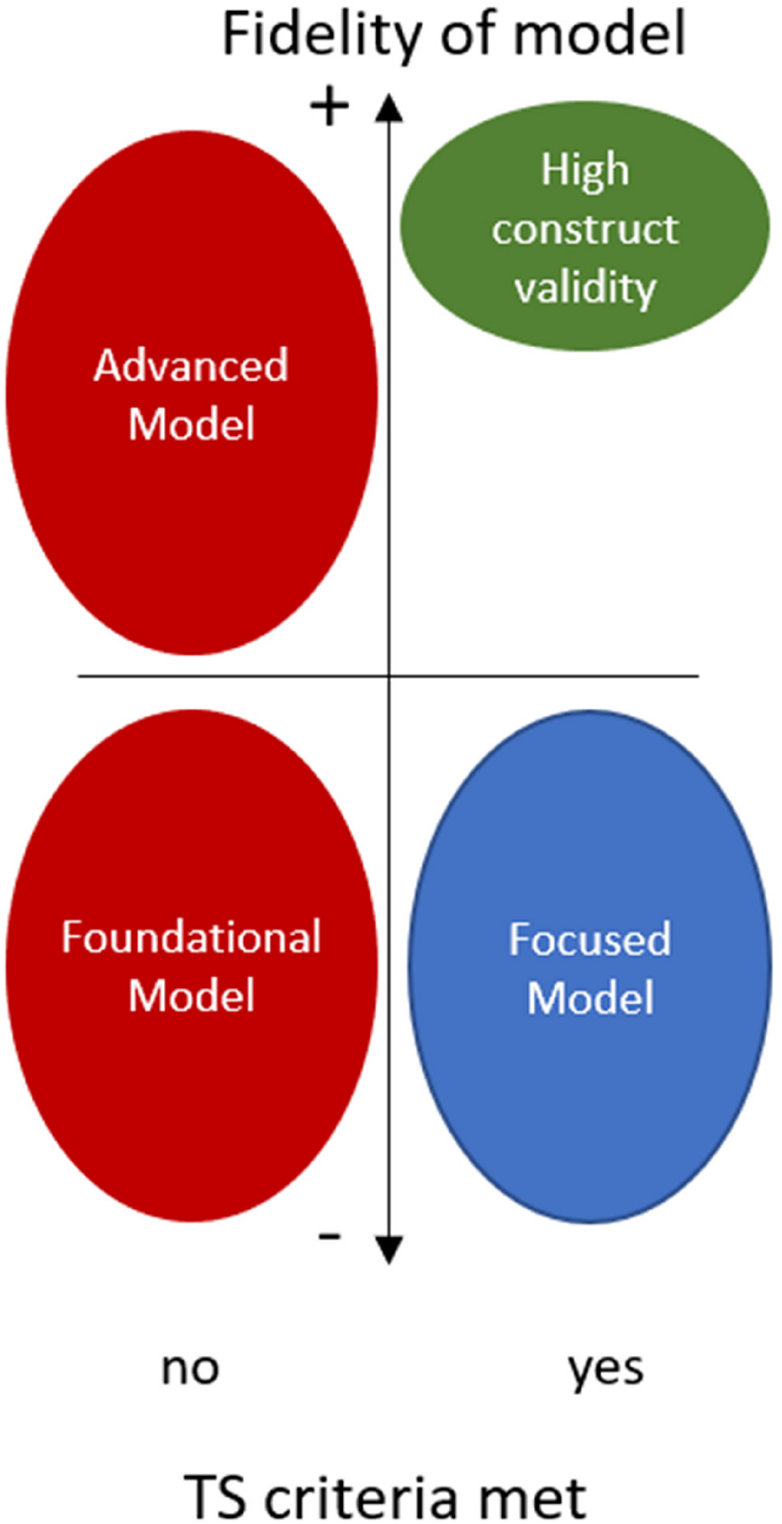
When employing models for conducting TS research, it's crucial that clinical TS criteria are met. A model that closely replicates other clinical features will enhance its translational possibilities (green). Study goals may justify reduced fidelity, but essential TS features should still be retained (blue). The majority of TS research lacks the incorporation of a proper definition (red). Advanced models do a good job in capturing human features but do not meet the criteria for TS. These models could offer insights or novel findings extending beyond the current clinical understanding. Foundational models are early-stage and basic models, typically in-vitro studies, that could provide essential insights or building blocks for TS understanding.

As we have discussed, the validity, reliability, and translational potential of an animal model are paramount. We have summarized TS features to assist researchers in this endeavour (Table 2). Table 3 presents a concise assessment tool for evaluating TS animal models. This tool incorporates the essential human TS features and considers favourable model-specific attributes. The first six features in the assessment tool correspond to the criteria and definition of TS in animal models. Maintaining high fidelity while achieving reproducibility is challenging. This is evident in the use of predisposed animals (gene-modified, pre-treated, specific age or sex, and so on) or in more accessible outcome assessments, which may impact construct validity to varying extents. Study goals may justify reduced fidelity, but essential TS features must be retained (Figure 3). Near-zero mortality rates are moreover ideal but challenging in diseases with inherent risk. Patients suffering from physically triggered TS have a 30-day mortality of 7–13.3% (40, 41), thus a model with no complications would limit its translational relevance.

**Table 3:** Assessment tool for animal models of Takotsubo syndrome.

^a^Clinically relevant trigger/experimental design
^a^Akinetic and/or dyskinetic regional wall motion abnormalities
^a^Acute onset
^a^Transient natural course
^a^Increased levels of cardiac biomarkers
^a^New ECG-changes
Genetically modified animal is not required
Low mortality
Reproducible

^a^ Definition and criteria for TS in animal models.

Any challenge to the body's homeostasis necessitates an adaptive response, engaging not only the cardiovascular system but also the nervous, endocrine, and immune systems. In the context of TS, triggers are broadly categorized as either emotional or physical, encompassing a wide array of stressors with variable responses. A model of TS should include an evaluation of the different physiological systems. This approach ensures a comprehensive understanding of the model, which can be achieved through imaging techniques, blood chemistry studies, histology, and various experimental setups. Myocardial infarction and myocarditis involve significant cardiac stress and may potentially act as triggers for TS by activating the body's stress response systems. However, it's critical to distinguish TS from these conditions, as TS is characterized by transient regional wall motion abnormalities without the coronary artery occlusion seen in myocardial infarction or the primary inflammation characteristic of myocarditis. Experimental designs aimed at modelling TS should employ triggers that replicate the stressors observed in human cases. This approach is intended to increase the likelihood that the model closely mirrors TS's pathophysiology and facilitates differentiation from myocardial infarction or myocarditis, based on the nature of the trigger and the subsequent cardiac response. We can further highlight pathophysiological distinctions or similarities by incorporating comparative studies, where animals exposed to different TS-specific triggers are contrasted with each other and those in established myocardial infarction or myocarditis models.

In addition to translational relevance, an animal model should prioritize reproducibility, low mortality, and minimal suffering, adhering to the principles of the 3Rs in animal research. Applying these principles to the study of TS, a condition closely linked with stress, deserves further consideration, though this topic extends beyond the scope of this article.

### 3.5 New and refined model of TS

We have recently introduced a refined TS model in outbred rats. This model exhibits high fidelity, demonstrating strong reproducibility (∼90%) at our facility, and low mortality (16.1%). Unlike the IP route, our model employs intravenous administration of isoprenaline at doses up to 100–500 times lower. This addresses the limitation of uncontrolled drug levels in the bloodstream associated with IP injections. Additionally, hyperthermic conditions (23) are unnecessary in this model, and it accurately replicates key TS features across sexes (86). The onset is acute, peaking at 4–6 h, followed by a recovery phase at 24 h. Rats recover in cardiac function and contractility within 1–14 days, with the majority recovering within 48–72 h. At 30 days, all surviving rats show normal behaviour, ECG, and imaging. Similar to human patients, the model replicates both a comparable complication profile and the significance of systolic cardiac function as a notable predictor for unfavourable outcomes. We are continuously evaluating the model, and so far, it demonstrates promising translational potential.

## 4 Conclusion

We advocate for use and definitions of TS in preclinical research to reflect the clinical setting, particularly within studies that implement animal models of TS. It is imperative to report the method for RWMA assessment and clearly state the TS definition employed in a study. The term “stress-induced cardiomyopathy” should be avoided and instead be viewed as a hypernym for TS. Studies using global function or cardiac injury alone to define TS should refrain from using terms like “TS,” “TS-like,” or “TS model.” Wall motion abnormality should be confined to akinesia or dyskinesia and measured using clinically applicable methods. Crucial features of TS necessary for its diagnosis should be present in a model of TS. A model that closely replicates other clinical features of TS will enhance its translational potential. Furthermore, considering the adaptive responses of the body to various stressors, a comprehensive evaluation of these physiological systems is essential for accurate interpretation. By using Embase and Pubmed for this literature review, we ensure a comprehensive coverage of the existing literature of animal models employed in TS research. Our in-depth analysis focused on animal models that consider key TS features, which also necessitated exclusion of non-English reports. Although this resulted in the exclusion of several articles and models studying various manifestations of stress, it considers the clinical picture, which is crucial for translational success. This article provides a guide for investigators conducting TS research with animals, enabling the successful translation of preclinical results into benefits for humans.
