# Supplementary material for: Animal models of Takotsubo syndrome: bridging the gap to the human condition
Source: Front Cardiovasc Med. 2024 May 22;11:1351587. doi: 10.3389/fcvm.2024.1351587 (PMC11152046; doi:10.3389/fcvm.2024.1351587)
Supplement: Supplementary file 1 [file Datasheet1.docx]

Supplementary

Search Strategy

References for this review were identified through PubMed and Embase searches until July 2023. The used MeSH term for takotsubo syndrome in PubMed was “Takotsubo Cardiomyopathy”, indexed both under “Cardiomyopathies” and “Ventricular dysfunction, left”. For the first search filter, Takotsubo Cardiomyoapthy was used as a [MeSH] term and from its listed entry terms we included additional [tiab] terms. For the second search block we used the search filter for finding all studies on animal experimentation on PubMed(1). The first and second search filters were then combined using AND so as to determine the number of animal studies on takotsubo. Note that this search is aimed to find models of TS, thus other articles relating the effects of stress and cardiac function might not be found, such as catecholamine-induced cardiac injury. The search filters were translated to be used in Embase as well. All identified records were uploaded to EndNote 20. A de-duplication process was applied before a first screen(2). During the screening process, the titles and abstracts of the references were examined. Only those references that showed no indication of studies related to animals and TS or stress-related studies, were removed from further consideration. The retrieved reports were subsequently screened for eligibility. As the diagnosis of Takotsubo syndrome (TS) is primarily based on the presence of regional wall motion abnormalities, only articles that included an assessment of this characteristic were included in the review. The record was excluded if it did not explicitly state this criterion. Additionally, publications written in languages other than English were not included in the review process.

**PubMed**

Search block 1

Stress cardiomyopathy[tiab] OR Takotsubo[tiab] OR Tako-tsubo[tiab] OR Apical ballooning syndrome[tiab] OR Transient-left-ventricular ballooning syndrome[tiab] OR Broken heart[tiab] OR "Takotsubo Cardiomyopathy"[Mesh]

Search block 2

"animal experimentation"[MeSH Terms] OR "models, animal"[MeSH Terms] OR "invertebrates"[MeSH Terms] OR "Animals"[Mesh:noexp] OR "animal population groups"[MeSH Terms] OR "chordata"[MeSH Terms:noexp] OR "chordata, nonvertebrate"[MeSH Terms] OR "vertebrates"[MeSH Terms:noexp] OR "amphibians"[MeSH Terms] OR "birds"[MeSH Terms] OR "fishes"[MeSH Terms] OR "reptiles"[MeSH Terms] OR "mammals"[MeSH Terms:noexp] OR "primates"[MeSH Terms:noexp] OR "artiodactyla"[MeSH Terms] OR "carnivora"[MeSH Terms] OR "cetacea"[MeSH Terms] OR "chiroptera"[MeSH Terms] OR "elephants"[MeSH Terms] OR "hyraxes"[MeSH Terms] OR "insectivora"[MeSH Terms] OR "lagomorpha"[MeSH Terms] OR "marsupialia"[MeSH Terms] OR "monotremata"[MeSH Terms] OR "perissodactyla"[MeSH Terms] OR "rodentia"[MeSH Terms] OR "scandentia"[MeSH Terms] OR "sirenia"[MeSH Terms] OR "xenarthra"[MeSH Terms] OR "haplorhini"[MeSH Terms:noexp] OR "strepsirhini"[MeSH Terms] OR "platyrrhini"[MeSH Terms] OR "tarsii"[MeSH Terms] OR "catarrhini"[MeSH Terms:noexp] OR "cercopithecidae"[MeSH Terms] OR "hylobatidae"[MeSH Terms] OR "hominidae"[MeSH Terms:noexp] OR "gorilla gorilla"[MeSH Terms] OR "pan paniscus"[MeSH Terms] OR "pan troglodytes"[MeSH Terms] OR "pongo pygmaeus"[MeSH Terms] OR animals[tiab] OR animal[tiab] OR mice[Tiab] OR mus[Tiab] OR mouse[Tiab] OR murine[Tiab] OR woodmouse[tiab] OR rats[Tiab] OR rat[Tiab] OR murinae[Tiab] OR muridae[Tiab] OR cottonrat[tiab] OR cottonrats[tiab] OR hamster[tiab] OR hamsters[tiab] OR cricetinae[tiab] OR rodentia[Tiab] OR rodent[Tiab] OR rodents[Tiab] OR pigs[Tiab] OR pig[Tiab] OR swine[tiab] OR swines[tiab] OR piglets[tiab] OR piglet[tiab] OR boar[tiab] OR boars[tiab] OR "sus scrofa"[tiab] OR ferrets[tiab] OR ferret[tiab] OR polecat[tiab] OR polecats[tiab] OR "mustela putorius"[tiab] OR "guinea pigs"[Tiab] OR "guinea pig"[Tiab] OR cavia[Tiab] OR callithrix[Tiab] OR marmoset[Tiab] OR marmosets[Tiab] OR cebuella[Tiab] OR hapale[Tiab] OR octodon[Tiab] OR chinchilla[Tiab] OR chinchillas[Tiab] OR gerbillinae[Tiab] OR gerbil[Tiab] OR gerbils[Tiab] OR jird[Tiab] OR jirds[Tiab] OR merione[Tiab] OR meriones[Tiab] OR rabbits[Tiab] OR rabbit[Tiab] OR hares[Tiab] OR hare[Tiab] OR diptera[Tiab] OR flies[Tiab] OR fly[Tiab] OR dipteral[Tiab] OR drosphila[Tiab] OR drosophilidae[Tiab] OR cats[Tiab] OR cat[Tiab] OR carus[Tiab] OR felis[Tiab] OR nematoda[Tiab] OR nematode[Tiab] OR nematoda[Tiab] OR nematode[Tiab] OR nematodes[Tiab] OR sipunculida[Tiab] OR dogs[Tiab] OR dog[Tiab] OR canine[Tiab] OR canines[Tiab] OR canis[Tiab] OR sheep[Tiab] OR sheeps[Tiab] OR mouflon[Tiab] OR mouflons[Tiab] OR ovis[Tiab] OR goats[Tiab] OR goat[Tiab] OR capra[Tiab] OR capras[Tiab] OR rupicapra[Tiab] OR chamois[Tiab] OR haplorhini[Tiab] OR monkey[Tiab] OR monkeys[Tiab] OR anthropoidea[Tiab] OR anthropoids[Tiab] OR saguinus[Tiab] OR tamarin[Tiab] OR tamarins[Tiab] OR leontopithecus[Tiab] OR hominidae[Tiab] OR ape[Tiab] OR apes[Tiab] OR pan[Tiab] OR paniscus[Tiab] OR "pan paniscus"[Tiab] OR bonobo[Tiab] OR bonobos[Tiab] OR troglodytes[Tiab] OR "pan troglodytes"[Tiab] OR gibbon[Tiab] OR gibbons[Tiab] OR siamang[Tiab] OR siamangs[Tiab] OR nomascus[Tiab] OR symphalangus[Tiab] OR chimpanzee[Tiab] OR chimpanzees[Tiab] OR prosimians[Tiab] OR "bush baby"[Tiab] OR prosimian[Tiab] OR bush babies[Tiab] OR galagos[Tiab] OR galago[Tiab] OR pongidae[Tiab] OR gorilla[Tiab] OR gorillas[Tiab] OR pongo[Tiab] OR pygmaeus[Tiab] OR "pongo pygmaeus"[Tiab] OR orangutans[Tiab] OR pygmaeus[Tiab] OR lemur[Tiab] OR lemurs[Tiab] OR lemuridae[Tiab] OR horse[Tiab] OR horses[Tiab] OR pongo[Tiab] OR equus[Tiab] OR cow[Tiab] OR calf[Tiab] OR bull[Tiab] OR chicken[Tiab] OR chickens[Tiab] OR gallus[Tiab] OR quail[Tiab] OR bird[Tiab] OR birds[Tiab] OR quails[Tiab] OR poultry[Tiab] OR poultries[Tiab] OR fowl[Tiab] OR fowls[Tiab] OR reptile[Tiab] OR reptilia[Tiab] OR reptiles[Tiab] OR snakes[Tiab] OR snake[Tiab] OR lizard[Tiab] OR lizards[Tiab] OR alligator[Tiab] OR alligators[Tiab] OR crocodile[Tiab] OR crocodiles[Tiab] OR turtle[Tiab] OR turtles[Tiab] OR amphibian[Tiab] OR amphibians[Tiab] OR amphibia[Tiab] OR frog[Tiab] OR frogs[Tiab] OR bombina[Tiab] OR salientia[Tiab] OR toad[Tiab] OR toads[Tiab] OR "epidalea calamita"[Tiab] OR salamander[Tiab] OR salamanders[Tiab] OR eel[Tiab] OR eels[Tiab] OR fish[Tiab] OR fishes[Tiab] OR pisces[Tiab] OR catfish[Tiab] OR catfishes[Tiab] OR siluriformes[Tiab] OR arius[Tiab] OR heteropneustes[Tiab] OR sheatfish[Tiab] OR perch[Tiab] OR perches[Tiab] OR percidae[Tiab] OR perca[Tiab] OR trout[Tiab] OR trouts[Tiab] OR char[Tiab] OR chars[Tiab] OR salvelinus[Tiab] OR "fathead minnow"[Tiab] OR minnow[Tiab] OR cyprinidae[Tiab] OR carps[Tiab] OR carp[Tiab] OR zebrafish[Tiab] OR zebrafishes[Tiab] OR goldfish[Tiab] OR goldfishes[Tiab] OR guppy[Tiab] OR guppies[Tiab] OR chub[Tiab] OR chubs[Tiab] OR tinca[Tiab] OR barbels[Tiab] OR barbus[Tiab] OR pimephales[Tiab] OR promelas[Tiab] OR "poecilia reticulata"[Tiab] OR mullet[Tiab] OR mullets[Tiab] OR seahorse[Tiab] OR seahorses[Tiab] OR mugil curema[Tiab] OR atlantic cod[Tiab] OR shark[Tiab] OR sharks[Tiab] OR catshark[Tiab] OR anguilla[Tiab] OR salmonid[Tiab] OR salmonids[Tiab] OR whitefish[Tiab] OR whitefishes[Tiab] OR salmon[Tiab] OR salmons[Tiab] OR sole[Tiab] OR solea[Tiab] OR "sea lamprey"[Tiab] OR lamprey[Tiab] OR lampreys[Tiab] OR pumpkinseed[Tiab] OR sunfish[Tiab] OR sunfishes[Tiab] OR tilapia[Tiab] OR tilapias[Tiab] OR turbot[Tiab] OR turbots[Tiab] OR flatfish[Tiab] OR flatfishes[Tiab] OR sciuridae[Tiab] OR squirrel[Tiab] OR squirrels[Tiab] OR chipmunk[Tiab] OR chipmunks[Tiab] OR suslik[Tiab] OR susliks[Tiab] OR vole[Tiab] OR voles[Tiab] OR lemming[Tiab] OR lemmings[Tiab] OR muskrat[Tiab] OR muskrats[Tiab] OR lemmus[Tiab] OR otter[Tiab] OR otters[Tiab] OR marten[Tiab] OR martens[Tiab] OR martes[Tiab] OR weasel[Tiab] OR badger[Tiab] OR badgers[Tiab] OR ermine[Tiab] OR mink[Tiab] OR minks[Tiab] OR sable[Tiab] OR sables[Tiab] OR gulo[Tiab] OR gulos[Tiab] OR wolverine[Tiab] OR wolverines[Tiab] OR minks[Tiab] OR mustela[Tiab] OR llama[Tiab] OR llamas[Tiab] OR alpaca[Tiab] OR alpacas[Tiab] OR camelid[Tiab] OR camelids[Tiab] OR guanaco[Tiab] OR guanacos[Tiab] OR chiroptera[Tiab] OR chiropteras[Tiab] OR bat[Tiab] OR bats[Tiab] OR fox[Tiab] OR foxes[Tiab] OR iguana[Tiab] OR iguanas[Tiab] OR xenopus laevis[Tiab] OR parakeet[Tiab] OR parakeets[Tiab] OR parrot[Tiab] OR parrots[Tiab] OR donkey[Tiab] OR donkeys[Tiab] OR mule[Tiab] OR mules[Tiab] OR zebra[Tiab] OR zebras[Tiab] OR shrew[Tiab] OR shrews[Tiab] OR bison[Tiab] OR bisons[Tiab] OR buffalo[Tiab] OR buffaloes[Tiab] OR deer[Tiab] OR deers[Tiab] OR bear[Tiab] OR bears[Tiab] OR panda[Tiab] OR pandas[Tiab] OR "wild hog"[Tiab] OR "wild boar"[Tiab] OR fitchew[Tiab] OR fitch[Tiab] OR beaver[Tiab] OR beavers[Tiab] OR jerboa[Tiab] OR jerboas[Tiab] OR capybara[Tiab] OR capybaras[Tiab]

**Embase**

Search block 1

'takotsubo cardiomyopathy'/exp OR 'stress cardiomyopathy':ti,ab OR takotsubo:ti,ab OR 'tako tsubo':ti,ab OR 'apical ballooning syndrome':ti,ab OR 'transient-left-ventricular ballooning syndrome':ti,ab OR 'broken heart':ti,ab OR 'ampulla syndrome':ti,ab

Search block 2

‘animal experiment’/exp OR ‘animal model’/exp OR ‘experimental animal’/exp OR ‘transgenic animal’/exp OR ‘male animal’/exp OR ‘female animal’/exp OR ‘juvenile animal’/exp OR animal/de OR chordata/de OR vertebrate/de OR tetrapod/de OR fish/exp OR amniote/de OR amphibia/exp OR mammal/de OR reptile/exp OR sauropsid/exp OR therian/de OR monotreme/exp OR ‘placental mammal’/de OR marsupial/exp OR Euarchontoglires/de OR Afrotheria/exp OR Boreoeutheria/exp OR Laurasiatheria/exp OR Xenarthra/exp OR primate/de OR Dermoptera/exp OR Glires/exp OR Scandentia/exp OR Haplorhini/de OR prosimian/exp OR simian/de OR tarsiiform/exp OR Catarrhini/de OR Platyrrhini/exp OR ape/de OR Cercopithecidae/exp OR hominid/de OR hylobatidae/exp OR chimpanzee/exp OR gorilla/exp OR ‘orangutan’/exp OR cephalopod/exp OR (animals OR animal OR mice OR mus OR mouse OR murine OR woodmouse OR rats OR rat OR murinae OR muridae OR cottonrat OR cottonrats OR hamster OR hamsters OR cricetinae OR rodentia OR rodent OR rodents OR pigs OR pig OR swine OR swines OR piglets OR piglet OR boar OR boars OR ‘sus scrofa’ OR ferrets OR ferret OR polecat OR polecats OR ‘mustela putorius’ OR ‘guinea pigs’ OR ‘guinea pig’ OR cavia OR callithrix OR marmoset OR marmosets OR cebuella OR hapale OR octodon OR chinchilla OR chinchillas OR gerbillinae OR gerbil OR gerbils OR jird OR jirds OR merione OR meriones OR rabbits OR rabbit OR hares OR hare OR diptera OR flies OR fly OR dipteral OR drosphila OR drosophilidae OR cats OR cat OR carus OR felis OR nematoda OR nematode OR nematoda OR nematode OR nematodes OR sipunculida OR dogs OR dog OR canine OR canines OR canis OR sheep OR sheeps OR mouflon OR mouflons OR ovis OR goats OR goat OR capra OR capras OR rupicapra OR chamois OR haplorhini OR monkey OR monkeys OR anthropoidea OR anthropoids OR saguinus OR tamarin OR tamarins OR leontopithecus OR hominidae OR ape OR apes OR pan OR paniscus OR ‘pan paniscus’ OR bonobo OR bonobos OR troglodytes OR ‘pan troglodytes’ OR gibbon OR gibbons OR siamang OR siamangs OR nomascus OR symphalangus OR chimpanzee OR chimpanzees OR prosimians OR ‘bush baby’ OR prosimian OR ‘bush babies‘ OR galagos OR galago OR pongidae OR gorilla OR gorillas OR pongo OR pygmaeus OR ‘pongo pygmaeus’ OR orangutans OR pygmaeus OR lemur OR lemurs OR lemuridae OR horse OR horses OR pongo OR equus OR cow OR calf OR bull OR chicken OR chickens OR gallus OR quail OR bird OR birds OR quails OR poultry OR poultries OR fowl OR fowls OR reptile OR reptilia OR reptiles OR snakes OR snake OR lizard OR lizards OR alligator OR alligators OR crocodile OR crocodiles OR turtle OR turtles OR amphibian OR amphibians OR amphibia OR frog OR frogs OR bombina OR salientia OR toad OR toads OR ‘epidalea calamita’ OR salamander OR salamanders OR eel OR eels OR fish OR fishes OR pisces OR catfish OR catfishes OR siluriformes OR arius OR heteropneustes OR sheatfish OR perch OR perches OR percidae OR perca OR trout OR trouts OR char OR chars OR salvelinus OR ‘fathead minnow’ OR minnow OR cyprinidae OR carps OR carp OR zebrafish OR zebrafishes OR goldfish OR goldfishes OR guppy OR guppies OR chub OR chubs OR tinca OR barbels OR barbus OR pimephales OR promelas OR ‘poecilia reticulata’ OR mullet OR mullets OR seahorse OR seahorses OR ‘mugil curema‘ OR ‘atlantic cod‘ OR shark OR sharks OR catshark OR anguilla OR salmonid OR salmonids OR whitefish OR whitefishes OR salmon OR salmons OR sole OR solea OR ‘sea lamprey’ OR lamprey OR lampreys OR pumpkinseed OR sunfish OR sunfishes OR tilapia OR tilapias OR turbot OR turbots OR flatfish OR flatfishes OR sciuridae OR squirrel OR squirrels OR chipmunk OR chipmunks OR suslik OR susliks OR vole OR voles OR lemming OR lemmings OR muskrat OR muskrats OR lemmus OR otter OR otters OR marten OR martens OR martes OR weasel OR badger OR badgers OR ermine OR mink OR minks OR sable OR sables OR gulo OR gulos OR wolverine OR wolverines OR minks OR mustela OR llama OR llamas OR alpaca OR alpacas OR camelid OR camelids OR guanaco OR guanacos OR chiroptera OR chiropteras OR bat OR bats OR fox OR foxes OR iguana OR iguanas OR ‘xenopus laevis‘ OR parakeet OR parakeets OR parrot OR parrots OR donkey OR donkeys OR mule OR mules OR zebra OR zebras OR shrew OR shrews OR bison OR bisons OR buffalo OR buffaloes OR deer OR deers OR bear OR bears OR panda OR pandas OR ‘wild hog’ OR ‘wild boar’ OR fitchew OR fitch OR beaver OR beavers OR jerboa OR jerboas OR capybara OR capybaras):ti,ab

1. Hooijmans CR, Tillema A, Leenaars M, Ritskes-Hoitinga M. Enhancing search efficiency by means of a search filter for finding all studies on animal experimentation in PubMed. Lab Anim. 2010;44(3):170-5.

2. Bramer WM, Giustini D, de Jonge GB, Holland L, Bekhuis T. De-duplication of database search results for systematic reviews in EndNote. J Med Libr Assoc. 2016;104(3):240-3.
